# Integrated metabolomics and machine learning identify predictive biomarkers via SHAP analysis for sintilimab-induced rash in lung cancer patients

**DOI:** 10.3389/fphar.2026.1846667

**Published:** 2026-06-03

**Authors:** Wenxiu Tian, Hehe Tang, Xiaoyuan Liu, Juan Lv, Yunyun Xu, Yujiao Hua, Fen Xie, Yongjuan Ding

**Affiliations:** Department of Clinical Pharmacy, Affiliated Hospital of Jiangnan University, Wuxi, Jiangsu Province, China

**Keywords:** differentially expressed metabolites, lung cancer, sintilimab, skin rash, untargeted metabolomics

## Abstract

**Background:**

Sintilimab-induced rash is a significant clinical challenge in lung cancer treatment, often necessitating therapy interruption or discontinuation and thereby compromising patient outcomes. The underlying mechanisms of this adverse event remain poorly understood. This study aimed to investigate potential predictive biomarkers and mechanisms of sintilimab-induced rash through metabolomic profiling.

**Methods:**

A total of 55 patients with lung cancer who received sintilimab were enrolled, including 32 who developed rash and 23 matched controls without rash. Blood samples were collected before sintilimab infusion and at rash onset. Comprehensive clinical data were recorded. Untargeted metabolomic analysis of plasma was performed using ultra-high-performance liquid chromatography–tandem mass spectrometry (UHPLC–MS/MS). Differential metabolites were identified and analyzed using pathway enrichment, univariate analysis (AUC ≥0.800), and SHAP analysis.

**Results:**

No significant differences were observed between groups in demographic characteristics or most clinical parameters. However, the rash group exhibited significantly elevated total bile acids glucose (GLU), and basophil percentage (BAS%), along with reduced AST/ALT ratio, alkaline phosphatase lactate dehydrogenase phosphorus (P), neutrophil count (NEU), and high-sensitivity C-reactive protein (hsCRP) (P < 0.05). Metabolomic analysis identified 92 differentially expressed metabolites. Pathway enrichment revealed alterations in oxytocin signaling, GnRH signaling, platelet activation, FcγR-mediated phagocytosis, retrograde endocannabinoid signaling, pantothenate and CoA biosynthesis, FcεRI signaling, and aldosterone synthesis and secretion. Univariate analysis identified 25 metabolites with high predictive value (AUC ≥0.800), and SHAP analysis highlighted 20 metabolites. Cross-comparison identified five overlapping metabolites: N,N,N-trimethyl-L-histidine, laurolactam, 2-naphthalenesulfonic acid, limonenecarboxylic acid, and N-lauroylsarcosine.

**Conclusion:**

Distinct clinical and metabolomic alterations are associated with sintilimab-induced rash in lung cancer patients. The identified differential metabolites may serve as predictive biomarkers and potential therapeutic targets, providing new insights for clinical management and mechanistic research into immune-related adverse events.

## 1 Introduction

Lung cancer remains a leading cause of global cancer mortality, with non-small cell lung cancer (NSCLC) accounting for approximately 85% of cases (Sung et al., 2021). Immune checkpoint inhibitors (ICIs), specifically PD-1/PD-L1 inhibitors such as sintilimab, have transformed NSCLC management by reactivating anti-tumor T-cell immunity, significantly enhancing survival in metastatic and locally advanced disease (Antonia et al., 2017). Sintilimab, a fully human anti–PD-1 IgG4 monoclonal antibody, showed efficacy in pivotal trials (e.g., ORIENT-11), establishing it as standard first-line therapy for PD-L1-positive NSCLC, administered alone or with chemotherapy (Yang et al., 2020; Zho et al., 2021). The widespread adoption of ICIs has shifted the therapeutic paradigm, leading to long-term treatment for an increasing number of NSCLC patients. However, their potent immunostimulatory effects trigger a distinct spectrum of inflammatory toxicities, termed immune-related adverse events (irAEs), posing significant clinical management challenges (Martins et al., 2019).

Cutaneous irAEs, particularly rash, are among the most frequent and problematic toxicities associated with sintilimab, affecting 15%–40% of patients, with severe (grade≥3) manifestations occurring in 1%–5% (Shi et al., 2019). This rash typically presents as pruritic, maculopapular eruptions on the trunk and extremities, emerging within weeks to months of treatment initiation (Lacouture et al., 2014). While mild cases are often managed with corticosteroids and temporary treatment holds, severe rash frequently necessitates permanent sintilimab discontinuation, potentially compromising anti-tumor efficacy and long-term survival (Dolladille et al., 2020). Notably, rash occurrence showed no consistent correlation with plasma sintilimab concentrations, rendering therapeutic drug monitoring ineffective for prediction or dose optimization (Zhao et al., 2017). Current management relies primarily on clinical assessment and immunosuppression, lacking validated predictive biomarkers or mechanism-based preventive strategies (Schneider et al., 2021). This critical gap underscores the urgent need to elucidate the pathophysiological mechanisms driving sintilimab-induced rash to enable proactive interventions.

The pathophysiology of ICI-induced rash remains incompletely elucidated, although dysregulated T-cell activation, cytokine release (e.g., IFN-γ, IL-17), and autoreactive antibody production are implicated (Alserawan et al., 2024; Watanabe and Yamaguchi, 2023). Genetic studies suggest certain HLA alleles (e.g., HLA-A*02:01, *HLA-DRB1*11:01) may confer susceptibility to severe cutaneous irAEs across ICI classes, though sintilimab-specific data are limited (Chowell et al., 2018). Furthermore, genomic approaches alone cannot fully account for phenotypic heterogeneity, indicating a role for dynamic, post-genomic factors like metabolic reprogramming (Johnson et al., 2016). Crucially, no studies have systematically investigated metabolic perturbations underlying sintilimab-associated rash. This represents a significant omission, as metabolites directly reflect functional cellular activity and immune cell energetics, potentially offering deeper mechanistic insights than genetic markers alone (Patti et al., 2012). From a pharmacological and network medicine perspective, understanding immune-metabolic reprogramming and pharmacotranscriptomic regulation is critical for revealing the mechanisms of immune-related adverse events (Tian et al., 2023a; Tian et al., 2023b).

Untargeted metabolomics provides a comprehensive, hypothesis-generating platform to profile the entire repertoire of low-molecular-weight metabolites (<1500 Da) within biological systems, capturing dynamic biochemical snapshots of physiological and pathological states (Wishart, 2016). Unlike genomics or proteomics, metabolomics detects rapid, downstream functional changes resulting from genetic, environmental, and therapeutic influences, revealing the functional endpoints of cellular processes (Beger et al., 2016). This approach has successfully uncovered metabolic signatures in diverse immune-mediated conditions, including psoriasis, atopic dermatitis, and checkpoint inhibitor-related colitis, identifying disruptions in pathways such as sphingolipid metabolism, tryptophan catabolism, and fatty acid oxidation (Gong et al., 2023). Applying untargeted metabolomics to sintilimab-induced rash holds substantial promise for discovering novel diagnostic biomarkers and revealing actionable therapeutic targets within dysregulated inflammatory or immune-modulatory pathways.

Given the high incidence, clinical burden, and mechanistic obscurity of sintilimab-induced rash in NSCLC patients, coupled with the absence of predictive tools or targeted interventions, innovative research approaches are imperative. Leveraging the proven capacity of metabolomics to decipher complex immune-toxicity mechanisms, this study aims to: (1) identify plasma metabolite profiles distinguishing NSCLC patients who develop sintilimab-induced rash from those without cutaneous toxicity using high-resolution mass spectrometry (HRMS)-based untargeted metabolomics; (2) characterize dysregulated metabolic pathways (e.g., eicosanoid, ceramide, amino acid metabolism); associated with rash pathogenesis; and (3) explore correlations between metabolite alterations and rash severity grades. We hypothesize that specific baseline or dynamic metabolic signatures predict rash susceptibility and that pathway analysis will implicate immune-dysregulatory mechanisms amenable to pharmacologic modulation. This foundational work seeks to provide critical insights for developing biomarker-driven prevention strategies and mechanism-based treatments, ultimately enhancing sintilimab safety and therapeutic continuity in lung cancer.

## 2 Materials and methods

### 2.1 Study population and data collection

This study enrolled lung cancer patients who received sintilimab for adjuvant therapy and were followed up at the Affiliated Hospital of Jiangnan University between December 2024 and December 2025. The inclusion criteria were as follows: (1) Patients with a pathological diagnosis of lung cancer; (2) Patients aged ≥18 years and ≤85 years; (3) All patients exhibited good medication adherence; (4) All patients were informed of the details of our study and understood the risks and benefits; (5) Life expectancy ≥12 weeks. The exclusion criteria were as follows: (1) Patients with prior exposure to any anti-PD-1, anti-PD-L1, anti-PD-L2, anti-CD137, or anti-CTLA-4 antibodies, or any other antibody or drug specifically targeting T-cell co-stimulation or checkpoint pathways; (2) Patients who developed rash prior to sintilimab administration; (3) Patients with severe hepatic and renal insufficiency; (4) Patients who received systemic treatment with Chinese herbal medicine with anti-tumor indications or immunomodulatory drugs (including thymosin, interferon, interleukin, *etc.*) within 2 weeks prior to the first dose; (5) Any other acute or chronic diseases, psychiatric disorders, or abnormal laboratory findings that could increase risk associated with study participation or study drug administration, or interference with the interpretation of study results, and in the investigator’s judgment, make the patient ineligible for participation in this study. The research protocol was sanctioned by the Ethics Committee of the Affiliated Hospital of Jiangnan University (LS2025022).

Through the review of electronic medical records, demographic data were collected, including parameters of liver and kidney function, thyroid function, and blood cell analysis.

### 2.2 Blood sample collection

Blood samples were collected before sintilimab infusion from patients in both groups. Whole blood (minimum 2 mL) was routinely drawn into EDTA anticoagulant tubes 22–24 h before drug administration. Within 1 h of collection, samples were centrifuged at 1620 *g* for 10 min at 4 °C. The resulting plasma was aliquoted into two portions and stored at −80 °C, designated for concentration measurement and metabolomics analysis respectively.

### 2.3 Untargeted metabolomic analysis

#### 2.3.1 Sample pretreatment

The plasma samples were thawed at 4 °C, vortex-mixed for 1 min to ensure homogeneity, and subsequently processed in a 96-well protein precipitation plate where 300 μL of 2-chlorophenylalanine solution (20 ppm, pre-stored at −20 °C) was added. Following the transfer of 50 μL aliquots of the sample to the plate, the mixture was vortexed for 5 min. The plate was then transferred to the A200 positive pressure nitrogen blowing module for 10 min under low positive pressure mode. After nitrogen concentration, the plate was sealed with a membrane in the designated testing area prior to LC-MS detection. For quality control, pooled QC samples were prepared by combining sample extracts, with analytical repeatability verified through overlay comparisons of total ion current (TIC) chromatograms across multiple QC injections (Demurtas et al., 2021).

#### 2.3.2 LC-MS/MS analysis

Analysis was performed using a Vanquish UHPLC system (Thermo Fisher Scientific, United States) coupled to an Orbitrap Exploris 120 mass spectrometer (Thermo Fisher Scientific, United States). Chromatographic separation was achieved on an ACQUITY UPLC® HSS T3 column (2.1 × 100 mm, 1.8 μm; Waters, United States) maintained at 40 °C with a flow rate of 0.3 mL/min and 5 μL injection volume. For ESI(+) mode, mobile phases consisted of (A_2_) 0.1% formic acid in water (v/v) and (B_2_) 0.1% formic acid in acetonitrile (v/v); for ESI(−) mode, phases were (A_3_) 5 mM ammonium formate and (B_3_) acetonitrile. Both modes employed identical gradient profiles: 0–1 min (10% B), 1–5 min (10%→98% B), 5–6.5 min (98% B), 6.5–6.6 min (98%→10% B), 6.6–8 min (10% B). The ESI source operated in dual-polarity mode with sheath gas 40 arb, aux gas 10 arb, capillary temperature 325 °C, spray voltage +3.50 kV (ESI+) and −2.50 kV (ESI-). Full MS acquisition covered *m/z* 100–1000 at 60,000 FWHM resolution, while ddMS^2^ mode performed data-dependent scans (4 per cycle) at 15,000 FWHM resolution with 30% normalized collision energy and automatic dynamic exclusion (Want et al., 2013).

#### 2.3.3 Quantification of sintilimab using a novel magnetic particle-based chemiluminescence immunoassay

This assay uses a novel chemiluminescence technique with high-quantum-yield acridinium ester labels and composite magnetic microparticles, in combination with anti-idiotypic antibodies, to achieve highly sensitive quantitative detection of sintilimab. The detailed procedure is as follows: 10 μL of sample was incubated with 50 μL of biotin-labeled anti-sintilimab antibody-2 (M-Biotin-Ab) for 5 min, followed by a washing step. Then, 50 μL of acridinium ester-labeled anti-sintilimab antibody-1 (AE-Ab) was added and allowed to react for another 5 min, after which three additional washes were performed. Finally, a trigger solution was introduced to initiate chemiluminescence detection. Throughout the process, the two antibodies form a sandwich immunocomplex with sintilimab present in the sample. Upon addition of streptavidin-conjugated magnetic microparticles, the complexes are captured onto the surface of the beads via specific biotin–streptavidin binding. After magnetic separation and washing to remove unbound components, the acridinium ester produces a luminescent signal under chemical excitation. The resulting luminescence intensity is proportional to the concentration of sintilimab in the sample, and the instrument automatically calculates the accurate concentration based on a pre-established standard curve.

#### 2.3.4 Data processing

Raw mass spectrometry data were converted to mzXML format using MSConvert within the ProteoWizard software package (v3.0.8789) (Rasmussen et al., 2022). Subsequent preprocessing—including feature detection, retention time correction, and peak alignment—was performed in R (v4.1.0) using the XCMS package (v3.12.0) (Navarro-Reig et al., 2015). Key parameters were configured as follows: ppm = 15, peakwidth = c (5, 30), mzdiff = 0.01, and method = centWave. Batch effects were mitigated by QC-sample-based correction. Metabolites exhibiting a relative standard deviation (RSD) > 30% in QC samples were excluded prior to downstream analysis.

Metabolite identification was achieved by matching accurate precursor ion mass (< 5 ppm) and MS/MS fragmentation patterns against the following databases: HMDB, MassBank, LipidMaps, mzCloud, KEGG, and a proprietary metabolite database (Panomix Biomedical Tech Co., Ltd., Suzhou, China). Molecular formulas were predicted using observed adduct ions and mass error tolerance. MS/MS spectra were validated against database reference fragments to confirm structural assignments.

### 2.4 Statistical analysis

The clinical characteristics of participants were assessed by Spearman correlation analysis in SPSS 25.0 software, and statistical significance was considered at *P* < 0.05. Multivariate dimensionality reduction analysis was performed on the sample data using the R package ropls (Thévenot et al., 2015), encompassing Principal Component Analysis (PCA), Partial Least Squares-Discriminant Analysis (PLS-DA), and Orthogonal PLS-DA (OPLS-DA). Potential model overfitting was assessed via permutation testing. Model performance was evaluated using R^2^X and R^2^Y (representing the explained variance of the X and Y matrices, respectively) and Q^2^ (predictive capability). Values approaching 1 indicated superior model goodness-of-fit and accurate classification of training-set samples into their original groups. Metabolites contributing to sample discrimination were screened based on statistical significance (*P* < 0.05), Variable Importance in Projection (VIP >1) derived from OPLS-DA, and fold change (FC) values reflecting inter-group abundance differences. Metabolites satisfying both VIP >1 and *P* < 0.05 were considered statistically significant discriminators. Pathway enrichment analysis was performed using a hypergeometric distribution-based approach (Xia and Wishart, 2011) to identify significantly enriched metabolic pathways. Resultant pathways were visualized via the KEGG Mapper tool, mapping differentially expressed metabolites onto canonical pathway diagrams.

### 2.5 Machine learning-based biomarker selection and validation

To identify the most impactful metabolic features, we employed SHAP (Shapley Additive explanations) analysis based on a Random Forest (RF) classification model (Koh et al., 2022). SHAP, a game theory-based approach, provides superior consistency over traditional feature importance metrics by calculating the mean absolute Shapley value for each feature across all samples, thereby yielding a robust ranking of global feature importance. The top 20 differentially expressed metabolites ranked by SHAP-based importance were selected as candidate biomarkers. These features were used to train and validate six distinct machine learning classifiers: K-Nearest Neighbors (KNN), Random Forest (RF), Support Vector Machine (SVM), Gaussian Naive Bayes (GNB), Logistic Regression (LR) and Decision Tree (DT). The dataset was partitioned into training and validation sets to ensure generalizability. The discriminatory power and efficacy of each classifier were rigorously assessed using receiver operating characteristic (ROC) curves. The area under the ROC curve (AUC) served as the primary metric for evaluating classification performance. Additional metrics, including accuracy, sensitivity (recall), and specificity, were also calculated to provide a comprehensive assessment of model performance.

## 3 Results

### 3.1 Basic characteristics of the participants

**FIGURE 1:**
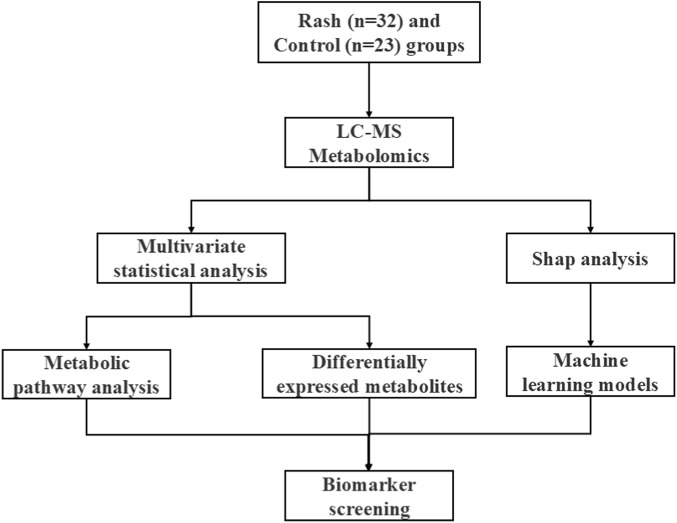
Overview of the study design.

The methodology employed in this study is shown schematically in Figure 1. This study enrolled 55 lung cancer patients receiving sintilimab adjuvant therapy, categorized into rash (n = 32) and non-rash (n = 23) groups. Baseline characteristics are presented in Table 1. No significant differences were observed between the two groups in terms of age, sex, cortisol, total bilirubin (TBIL), direct bilirubin (DBIL), indirect bilirubin (IBIL), albumin (ALB), prealbumin (PA), alanine aminotransferase (ALT), aspartate aminotransferase (AST), mitochondrial AST (ASTm), gamma-glutamyl transferase (GGT), creatine kinase (CK), adenosine deaminase (ADA), urea, cystatin C (CYCS), free triiodothyronine (FT3), free thyroxine (FT4), thyroid-stimulating hormone (TSH), white blood cell count (WBC), red blood cell count (RBC), hemoglobin (HGB), platelet count (PLT), mean corpuscular volume (MCV), mean corpuscular hemoglobin (MCH), or red cell distribution width-coefficient of variation (RDW-CV) (*P* > 0.05 for all). However, the rash group showed significantly elevated levels of total bile acids (TBA), glucose (GLU), and basophil percentage (BAS%) (*P* < 0.05), alongside lower AST/ALT ratio, alkaline phosphatase (ALP), lactate dehydrogenase (LDH), phosphorus (P), neutrophil count (NEU), and high-sensitivity C-reactive protein (hsCRP) (*P* < 0.05) compared to the non-rash group.

**TABLE 1:** Patients characteristics.

	Control (n = 23)	Rash (n = 32)	P.value
Gender (male/female)	16/7	26/6	0.257
Age (years)	66.28 (56.00-76.00)	68.88 (42.00-78.00)	0.069
Dose of imatinib (mg/day)	200	200	-
Concentration of sindilimab (μg/mL)	31.45 (5.74-60.19)	23.41 (0.84-52.15)	0.055
AST/ALT (U/L)	1.91 (0.43-3.64)	1.44 (0.44-3.40)	0.007**
ALP (U/L)	105.78 (52.00-255.00)	76.94 (52.00-128.00)	0.001**
LDH (U/L)	269.09 (153.00-862.00)	183.34 (114.00-293.00)	0.000**
TBA (umol/L)	6.39 (2.10-19.90)	8.59 (2.80-28.50)	0.017*
GLU (umol/L)	5.20 (3.86-8.26)	5.80 (3.84-9.05)	0.037*
P (umol/L)	1.23 (0.77-1.54)	1.14 (0.80-1.58)	0.019*
CORTISO (ug/dL)	10.83 (0.95-29.01)	8.42 (0.09-19.64)	0.309
NEU (10-9/L)	4.49 (2.30-8.00)	3.69 (1.20-13.50)	0.041*
BAS%	0.30 (0.10-0.80)	0.45 (0.10-1.40)	0.022*
hsCRP (mg/L)	55.21 (0.70-349.70)	6.71 (0.30-50.60)	0.009**
TBIL (umol/L)	11.23 (5.00-33.30)	10.20 (5.10-23.10)	0.567
DBIL (umol/L)	2.11 (0.80-5.90)	1.96 (0.80-4.30)	0.826
IBIL (umol/L)	9.12 (3.80-27.40)	8.24 (4.30-18.80)	0.495
ALB (g/L)	35.36 (24.40-45.30)	36.06 (27.70-42.40)	0.717
PA (g/L)	206.43 (86.20-314.70)	213.77 (64.50-369.90)	0.960
ALT (U/L)	34.17 (4.00-337.00)	20.97 (5.00-81.00)	0.723
AST (U/L)	65.09 (8.00-776.00)	25.25 (9.00-79.00)	0.088
ASTm (U/L)	20.94 (4.70-247.00)	8.71 (2.80-20.80)	0.130
GGT (U/L)	38.61 (15.00-96.00)	40.22 (11.00-116.00)	0.973
CK (U/L)	77.78 (12.00-411.00)	56.25 (9.00-135.00)	0.933
ADA (U/L)	12.53 (6.50-38.70)	11.23 (4.30-17.90)	0.987
Urea (mmol/L)	6.60 (2.39-19.02)	5.61 (3.26-11.05)	0.290
CYCS (mg/L)	1.15 (0.72-2.44)	1.15 (0.68-2.35)	0.595
FT3 (pg/mol)	2.51 (1.34-3.10)	2.53 (1.30-3.15)	0.814
FT4 (ng/dL)	1.06 (0.59-1.55)	1.04 (0.84-1.53)	0.299
TSH (mlU/L)	2.56 (0.50-6.87)	2.53 (0.01-12.35)	0.743
WBC (10-9/L)	6.30 (4.10-9.30)	5.71 (3.30-15.50)	0.070
RBC (10-12/L)	3.84 (1.85-4.79)	3.72 (2.46-5.53)	0.293
HGB (g/L)	115.81 (59.00-145.00)	113.77 (63.00-137.00)	0.692
PLT (10-9/L)	238.90 (88.00-386.00)	210.07 (100.00-381.00)	0.224
MCV (fl)	93.50 (83.20-104.80)	93.74 (79.40-103.50)	0.806
MCH (pg)	30.30 (26.60-32.90)	30.68 (24.70-34.00)	0.320
RDW-CV (%)	15.00 (12.50-19.70)	14.45 (12.50-19.10)	0.514

Abbreviations: AST/ALT: aspartate aminotransferase/alanine aminotransferase; ALP: alkaline phosphatase; LDH: lactate dehydrogenase; TBA: total bile acids; GLU: glucose; P: phosphorus; CORTISO: cortisol; NEU#: absolute neutrophil count; BAS%: basophil percentage; hsCRP: high-sensitivity C-reactive protein; TBIL: total bilirubin; DBIL: direct bilirubin; IBIL: indirect bilirubin; ALB: albumin; PA: prealbumin; ALT: alanine aminotransferase; AST: aspartate aminotransferase; ASTm: mitochondrial isoenzyme of AST; GGT: gamma-glutamyl transferase; CK: creatine kinase; ADA: adenosine deaminase; Urea: urea; CYCS: cystatin C; FT3: free triiodothyronine; FT4: free thyroxine; TSH: thyroid-stimulating hormone; WBC: white blood cell count; RBC: red blood cell count; HGB: hemoglobin; PLT: platelet count; MCV: mean corpuscular volume; MCH: mean corpuscular hemoglobin; RDW-CV: red cell distribution width-coefficient of variation.

Statistical significance: **Correlation is significant at the 0.01 level (2-tailed). *Correlation is significant at the 0.05 level (2-tailed).

### 3.2 Detection of endogenous metabolites in plasm

**FIGURE 2:**
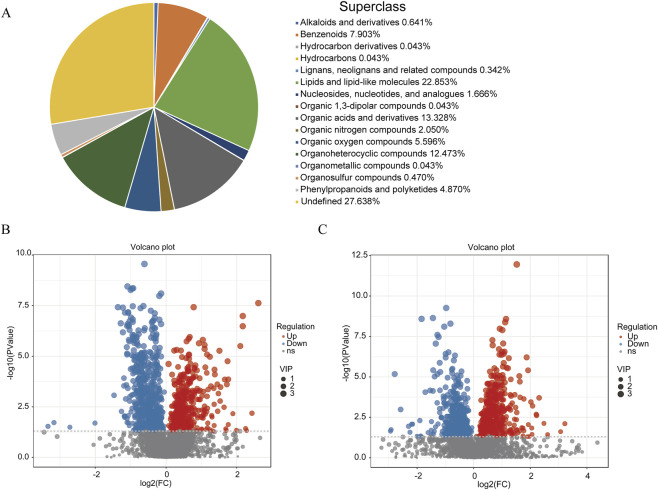
The number proportion of identified metabolites in each chemical classification **(A)** and volcano map in positive mode detection **(B)** and negative mode detection **(C)**. Significantly differentially expressed metabolites: meet FC > 1.5 and p < 0.05 are shown in red, meet FC < 0.67 and p < 0.05 are shown in blue. Non-significantly differentially expressed metabolites are shown in black.

In mass spectrometry-based metabolomics, principal component analysis (PCA) is widely used to assess analytical quality (Dunn et al., 2011). The tight clustering of quality control (QC) samples in PCA score plots reflects sustained system stability over the entire experimental workflow (Supplementary Figure S1). Although QC samples are nominally identical, technical variations introduced during sample preparation and instrumental analysis can lead to minor deviations. A high degree of clustering among QC samples indicates strong analytical robustness, thereby affirming the reliability of the resulting data. Untargeted LC-MS/MS metabolomic profiling detected 2,341 compounds, with 1275 and 1066 metabolites identified in positive and negative ionization modes, respectively. These metabolites spanned 16 chemical classes (Figure 2A), with their distribution detailed in Figure 2A. Volcano plot analysis revealed significant differentially expressed metabolites between control and rash groups (VIP >1 and *P* < 0.05) in both ionization modes (Figures 2B,C).

### 3.3 Identification of differential metabolites between control group and rash group

**FIGURE 3:**
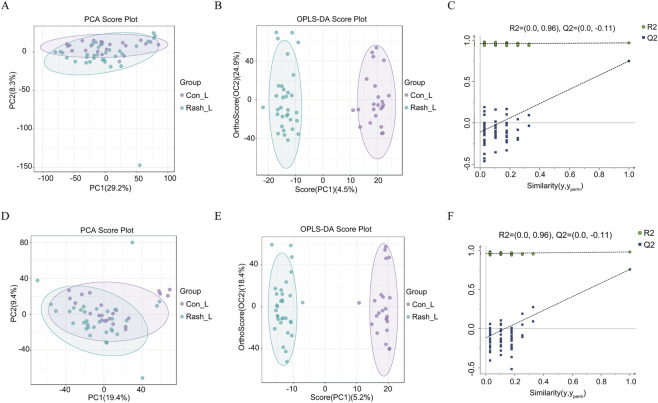
Multivariate modelling of LC-MS data after log transformation and pareto scaling. **(A)** PCA score plot of LC-MS (+) data: R^2^X = 0.52; **(B)** OPLS-DA score plot of LC-MS (+) data: R^2^X = 0.449, *R* ^2^ Y = 0.968, Q^2^ = 0.749; **(C)** the 200-permutation test of LC-MS (+) data; **(D)** PCA score plot of LC-MS (−) data: R^2^X = 0.508; **(E)** OPLS-DA score plot of LC-MS (−) data: R^2^X = 0.36, R^2^Y = 0.981, Q^2^ = 0.758; **(F)** the 200-permutation test of LC-MS (−) data.

Principal component analysis (PCA) and orthogonal partial least squares-discriminant analysis (OPLS-DA) score plots demonstrated clear metabolic separation between control and rash groups in both positive (Figures 3A,B) and negative (Figures 3D,E) ionization modes. Permutation testing (n = 200) validated model robustness, where all permuted Q^2^ values remained below their original counterparts with a negative intercept (Q^2^ < 0.5; Figures 3C,F), confirming absence of overfitting. These results establish the model’s predictive reliability for group discrimination.

**FIGURE 4:**
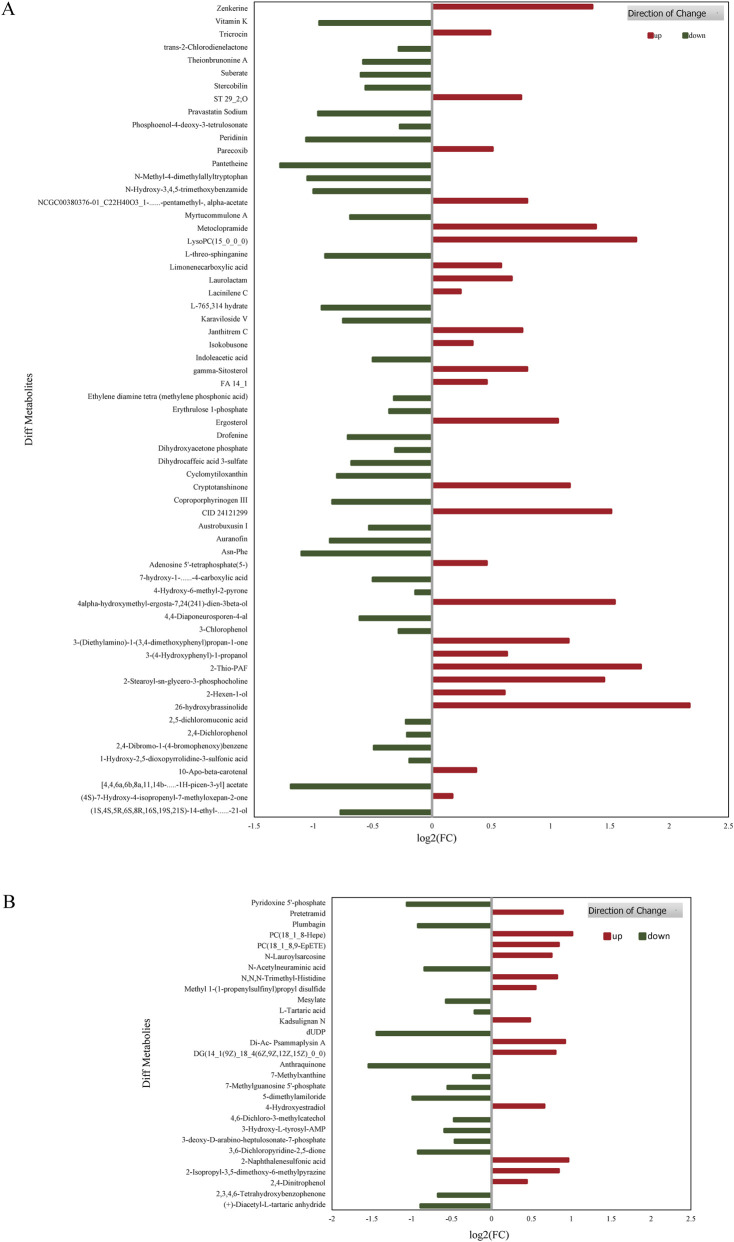
Fold change analysis of significantly differentially expressed metabolites in positive mode detection **(A)** and negative mode detection **(B)**. Red indicates upregulated and green indicates downregulated.

To identify biologically significant differentially expressed metabolites, we applied dual criteria of Variable Importance in Projection (VIP >2) and statistical significance (*P* < 0.05). This approach identified 92 differentially expressed metabolites: 63 in positive mode (27 upregulated, 36 downregulated) and 29 in negative mode (13 upregulated, 16 downregulated) (Supplementary Table S1). The chemical classification of these metabolites was visualized in a histogram color-coded by structural class (Figure 4).

### 3.4 Functional analysis of differentially expressed metabolites

To evaluate the metabolic correlations among the screened differentially expressed metabolites, we performed pairwise correlation analysis using Pearson correlation coefficients (PCC). This analysis evaluates the consistency of metabolite variation patterns. Metabolites exhibiting synergistic relationships showed concordant directional changes (positive correlation, PCC approaches +1), while antagonistic relationships manifested as discordant trends (negative correlation, CC approaches −1). Correlations were considered statistically significant at *P* < 0.05 (Rao et al., 2016). The results are visualized in correlation heatmaps (Supplementary Figure S2). Several metabolite pairs exhibited strong correlations, including: 5-Guanidino-3-methyl-2-oxopentanoic acid/Acetyl-L-Carnitine, Methyl dihydrojasmonate/(S,E)-Lyratol propanoate, MG (0_0_18_3 (6Z,9Z,12Z)_0_0)/5,6-dihydroxy-8Z,11Z, 14Z-eicosatrienoic acid, Dolichyl D-xylosyl phosphate/Eriocarpin, Linamarin/L-Rhamnose, Benzyl sulfate/3,4-Dimethoxycinnamic acid, Lignicol/N5,N5-dimethyl-4-nitro-2, 3-benzothiadiazol-5-amine, 3,4-Dimethoxycinnamic acid/5-Guanidino-3-methyl-2-oxopentanoic acid, Ligusticide/Citral propylene glycol acetal, Epithienamycin E/Cyclic glycerate-2,3P2, 3,4-Dimethoxycinnamic acid/Acetyl-L-Carnitine.

**FIGURE 5:**
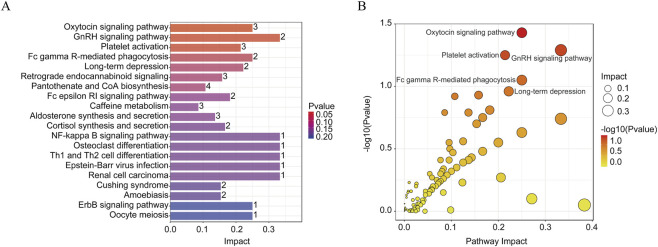
Metabolic pathway enrichment analysis. **(A)** Histogram. **(B)** Bubble diagram.

Pathway enrichment analysis of significant metabolites was conducted using the KEGG database. Related pathways could be classified into Oxytocin signaling pathway, GnRH signaling pathway, Platelet activation, Fc gamma R-mediated phagocytosis, Long-term depression, Retrograde endocannabinoid signaling, Pantothenate and CoA biosynthesis, Fc epsilon RI signaling pathway, Caffeine metabolism, Aldosterone synthesis and secretions (Figure 5).

### 3.5 Diagnostic performance and machine learning analysis

Receiver operating characteristic (ROC) curve analysis was performed to assess the diagnostic potential of the differentially expressed metabolites. The area under the curve (AUC) was used to evaluate the diagnostic accuracy of prospective biomarkers identified through univariate analysis. This analysis revealed 25 metabolites exhibiting significant predictive value for distinguishing the rash group from the control group (AUC ≥0.800; Supplementary Table S2).

**FIGURE 6:**
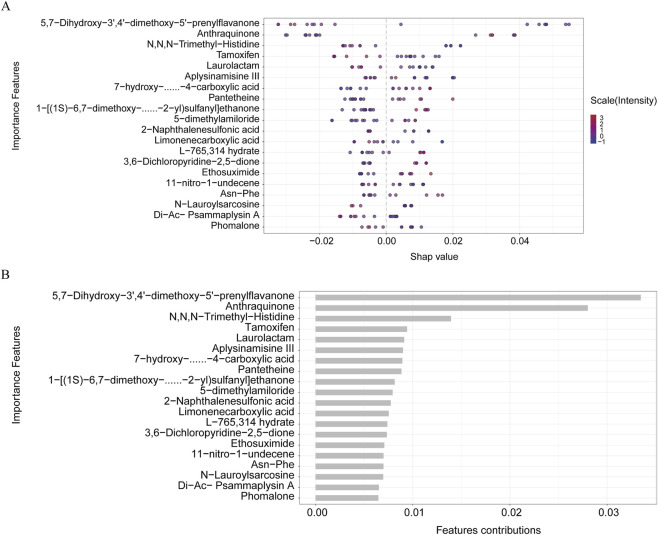
Feature importance analysis. **(A)** Feature importance ranking. **(B)** Beeswarm plot of important features.

**FIGURE 7:**
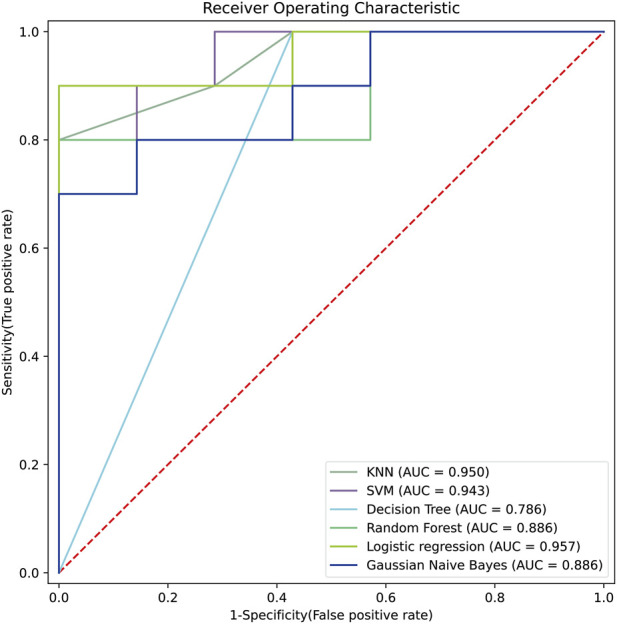
ROC curves illustrating the prediction performance of multiple models.

SHAP analysis effectively quantified the contribution of individual metabolite features to the predictions of the Random Forest model, producing a global ranking of feature importance. The direction and magnitude of the predictive impact of the top 20 most influential metabolites are clearly shown (Figure 6A). In addition, we present the ranking of these metabolites based on their contribution to model classification (Figure 6B). This set of 20 candidate biomarkers, selected based on SHAP-based importance, was used to train six distinct machine learning models. All classifiers demonstrated strong predictive performance for patient outcomes, as indicated by their ROC curves and corresponding AUC values (Figure 7). The AUC values approached 1.0, reflecting excellent discriminatory power accompanied by high sensitivity and specificity. This analysis confirms that the identified metabolite panel constitutes a robust and informative signature for classification, as it consistently supported high predictive accuracy across diverse machine learning algorithms. Moreover, although multiple classifiers consistently yielded high AUC values close to 1.0, this may partly reflect overfitting to the relatively small dataset; rigorous external validation in fully independent cohorts is essential to confirm the real-world predictive performance of the identified biomarker panel.

## 4 Discussion

Skin rash was a frequent adverse event among patients with non-small cell lung cancer (NSCLC) who received sintilimab, necessitating early detection to mitigate treatment discontinuation risk and improve outcomes. As the first untargeted metabolomics study investigating sintilimab-induced cutaneous toxicity in NSCLC, we identified significant plasma metabolic alterations between rash-affected patients and matched controls. Pathway analysis revealed perturbations in oxytocin signaling, GnRH signaling, platelet activation, FcγR-mediated phagocytosis, retrograde endocannabinoid signaling, pantothenate and CoA biosynthesis, FcεRI signaling, and aldosterone synthesis/secretion. Univariate analysis (AUC ≥ 0.800) identified 25 significantly differentially expressed metabolites, while SHAP analysis selected 20 metabolites with substantial predictive value. Cross-comparison between these two metabolite panels identified five overlapping metabolites: N,N,N-trimethyl-L-histidine, laurolactam, 2-naphthalenesulfonic acid, limonenecarboxylic acid, and N-lauroylsarcosine. These co-identified metabolites offer novel insights into the pathogenesis of immune checkpoint inhibitor-related skin toxicity and represent promising candidates for further biomarker development.

To investigate the clinical context of these metabolic disturbances, we analyzed a cohort of lung cancer patients with a median age of 60 years (range: 42–78 years). Neither age (*P* = 0.069) nor sex distribution (*P* = 0.257) differed significantly between patients with and without cutaneous rash. Nevertheless, the observed trend toward a younger age in the rash group, though not statistically significant, suggests that age might be a modifying factor deserving further exploration in larger-scale studies. Current clinical studies and real-world evidence indicate that the development of sintilimab-associated rash lacks a clear direct correlation with drug dosage or plasma concentration levels (Shi et al., 2022). The mechanism is primarily attributed to immune hyperactivation resulting from PD-1/PD-L1 pathway blockade, which leads to autoimmune-like reactions against normal tissues such as the skin, categorizing it as an immune-related adverse event (irAE) (Postow et al., 2018). This response is influenced more by individual variations in immune reactivity than by the conventional “dose-dependent toxicity” profile commonly observed with cytotoxic chemotherapeutic agents. Consequently, the emergence of rash is more closely associated with the extent of immune activation rather than attainment of a specific drug exposure threshold. Supporting this, clinical investigations of other PD-1 inhibitors, including nivolumab (Topalian et al., 2012), have similarly reported no significant association between rash incidence or severity and administered dose or systemic drug concentrations.

Notably, patients with rash exhibited distinct biochemical alterations compared to those without rash. Specifically, the rash group had significantly higher levels of total bile acids (TBA), glucose (GLU), and basophil percentage (BAS%) (P < 0.05). Conversely, they showed significantly lower AST/ALT ratio, alkaline phosphatase (ALP), lactate dehydrogenase (LDH), phosphorus (P), neutrophil count (NEU), and high-sensitivity C-reactive protein (hsCRP) (P < 0.05). Our data implicate sintilimab-induced rash in a multifactorial immunoinflammatory cascade. Mechanistically, the significantly elevated BAS% suggests Th2-polarized immunity, aligning with immune checkpoint inhibitor (ICI)-induced atopic-like cutaneous reactions (Sibaud, 2018). The reduced NEU indicates a non-neutrophilic inflammatory pattern, consistent with reported hematologic irAEs in ICI therapy (Yerolatsite et al., 2024). Markedly increased TBA may activate the pruritogenic receptor TGR5 on cutaneous sensory neurons, directly promoting itch and barrier dysfunction (Lieu et al., 2014), while hyperglycemia exacerbates skin inflammation through the AGE-RAGE axis, as demonstrated in inflammatory dermatoses (Ikumi et al., 2019). Collectively, these alterations pointed to concurrent hepatobiliary injury, metabolic dysregulation, and aberrant Th2 immunity as multifactorial drivers of sintilimab-related cutaneous toxicity.

To elucidate the molecular pathways underpinning these observed clinical and biochemical changes, we performed KEGG enrichment analysis of the altered metabolites. This analysis revealed that the differentially expressed metabolites were significantly enriched in pathways critical for immune effector functions, inflammatory signaling, and metabolic regulation pertinent to cutaneous immunity. These included platelet activation, FcγR-mediated phagocytosis, retrograde endocannabinoid signaling, pantothenate and CoA biosynthesis, FcεRI signaling, and aldosterone synthesis and secretion. Platelet activation and FcγR-mediated phagocytosis pathways facilitate the clearance of opsonized targets and initiate inflammatory responses; their dysregulation may contribute to immune-mediated tissue injury (Nieswandt et al., 2011). Notably, the FcεRI signaling pathway directly regulates mast cell degranulation, a key pathogenic event in drug-induced skin rashes involving histamine release and urticaria. Concurrently, perturbations in retrograde endocannabinoid signaling could disrupt cutaneous neuro-immune homeostasis, potentially modulating inflammatory thresholds in the skin (Liu et al., 2021). Furthermore, pantothenate and CoA biosynthesis, essential for acyl carrier protein function, fatty acid metabolism, and cellular energy status, influences inflammatory mediator production; its impairment may compromise epidermal barrier repair (Miallot et al., 2023). Dysregulated aldosterone synthesis and secretion might also exacerbate inflammatory edema by affecting electrolyte balance and vascular permeability within the dermal microenvironment (Herrada et al., 2011). These metabolic and immune pathway disturbances are consistent with recent pharmacological findings linking PD-1 inhibition, metabolic reprogramming, and cancer therapeutic responses (Tian et al., 2025a; Tian et al., 2026). Collectively, the enrichment of these pathways suggests a multifaceted interplay between sintilimab-induced immunomodulation, altered effector cell functions (particularly involving Fc receptors and mast cells), dysregulation of neuro-immune-endocrine crosstalk, and impaired metabolic support, collectively contributing to the development of cutaneous adverse reactions, including rash.

Among the differentially expressed metabolites contributing to these pathway perturbations, we focused on N,N,N-trimethyl-L-histidine, more commonly known as ergothioneine, given its potential link to histaminergic signaling and the FcεRI pathway. This muscle-derived metabolite was significantly elevated in NSCLC patients who developed an immune-related rash following sintilimab treatment, highlighting a potential link between PD-1 inhibitor-induced immunometabolic reprogramming and cutaneous toxicity. Sintilimab-enhanced T-cell activation promotes skin infiltration of Th1/Th17 cells and mast cell degranulation via cytokines including IL-3, IL-31, and GM-CSF, leading to histamine release and classic rash symptoms such as vasodilation, edema, and pruritus (Watanabe and Yamaguchi, 2023; Yamamoto, 2022). The accumulation of ergothioneine, a methylated histidine derivative, reflects systemic reprogramming of histamine metabolism, likely indicating impaired degradation through histamine-N-methyltransferase (HNMT) and a histamine-driven hyperinflammatory state (Hamid and Hamidi, 2024). This methylated amino acid dysregulation may arise from immune-induced hyperactivity of S-adenosylmethionine metabolism and keratinocyte stress responses (Chen et al., 2023). Notably, ergothioneine can activate the NLRP3 inflammasome to promote IL-1β release and directly aggravate skin inflammation (Higashikuni et al., 2023); its structural similarity to histamine also suggests potential competitive binding at H1 receptors, likely modulating pruritus signaling. Furthermore, clonal expansion of activated T cells heightens dependence on amino acid availability, potentially disturbing muscle metabolite homeostasis and facilitating release of endogenous peptides including carnosine derivatives into circulation (Ma et al., 2024; Reschke et al., 2022). Histamine may also enhance local dendritic cell activation, amplify T-cell responses and create a pro-inflammatory feedback loop that sustains skin inflammation (Liu et al., 2023). Thus, ergothioneine may serve not only as a biomarker of histamine pathway dysregulation in sintilimab-induced rash, but also play a functional role in linking immune-mediated amino acid metabolism and cutaneous inflammatory responses.

Another differentially abundant metabolite identified in this study is laurolactam, a 12-membered ring lactam structurally associated with fatty acid metabolism and potentially originating from the cyclization of long-chain fatty acids or amino acids *in vivo* (Fukuta et al., 2009). Notably, its structural similarity to Azone (laurocapsome), a known skin penetration enhancer that disrupts stratum corneum lipid organization to promote transdermal drug delivery (Fukuta et al., 2009), leads us to hypothesize that accumulated laurolactam may act as an endogenous penetration enhancer under sintilimab treatment, facilitating the entry of drug components or inflammatory mediators into the skin and thereby contributing to rash initiation. Moreover, the skin barrier relies on a lipid matrix comprising ceramides, cholesterol, and free fatty acids (Wertz, 2021). Sintilimab-induced systemic T-cell immune activation and release of pro-inflammatory cytokines (e.g., TNF-α, IFN-γ, IL-17, IL-22) can directly inhibit the expression of key lipid metabolic enzymes (such as serine palmitoyl transferase and β-glucocerebrosidase) in keratinocytes, resulting in disrupted epidermal lipid homeostasis (Martins et al., 2020). As both a marker and potential functional contributor to this metabolic dysregulation, elevated laurolactam may not only signify barrier impairment but also further destabilize lipid architecture, increase trans epidermal water loss, and facilitate the invasion of foreign allergens and microorganisms. These effects could synergistically enhance local immune responses and T-cell infiltration, ultimately driving severe and persistent inflammatory rash (Gooran et al., 2023).

In addition to endogenous metabolites, we also observed significant alteration of 2-naphthalenesulfonic acid, an exogenous aromatic sulfonic acid frequently found in dyes and chemical intermediates, which is primarily derived from the sulfonation of pharmaceuticals or environmental chemicals *in vivo* (Cao et al., 2023). Its pronounced elevation suggests that sintilimab therapy may perturb host metabolic homeostasis, potentially through dysregulation of drug-metabolizing enzymes and gut microbial imbalance. Growing evidence links immune-related adverse events induced by checkpoint inhibitors to alterations in gut microbiota composition; specific dysbiosis may facilitate the overgrowth of bacteria capable of generating aromatic metabolites such as 2-naphthalenesulfonic acid, promoting its systemic accumulation (Pezo et al., 2019). This compound can translocate to the skin, where it may behave as a hapten by covalently binding cutaneous proteins (e.g., keratins) to form neoantigens. Under conditions of skin barrier impairment—often associated with T-cell-mediated inflammation—these neoantigens can be captured by antigen-presenting cells and presented to sintilimab-primed T cells, potentially eliciting a delayed-type (type IV) hypersensitivity reaction that amplifies rash initiation and progression (Zemelka-Wiacek et al., 2022). Furthermore, 2-naphthalenesulfonic acid possesses documented antioxidant and antimicrobial properties that may modulate the local redox balance and microbial composition in the skin, thereby indirectly influencing inflammatory pathways (Mehra and Chadha, 2020). Thus, the accumulation of this metabolite not only signifies aberrant processing of exogenous compounds during treatment but may also actively contribute to the immunopathological mechanisms underlying cutaneous adverse reactions.

Similarly, limonene carboxylic acid—an oxidized metabolite of limonene, a monoterpene abundant in citrus fruits—can be generated through both endogenous metabolic pathways and atmospheric oxidation processes (Wang and Wang, 2021; Rinaldi de Alvarenga et al., 2022). Although limonene itself possesses low sensitization potential, its oxidation derivatives, including limonene hydroperoxide and limonene carboxylic acid, are recognized as potent contact allergens (Bennike et al., 2019). Notably, this metabolite has also been associated with anti-inflammatory, antioxidant, and immunomodulatory activities, such as suppression of the NF-κB pathway, reduction of pro-inflammatory cytokines (e.g., TNF-α and IL-6), and elevation of anti-inflammatory IL-10 (Kathem et al., 2024). Within the setting of sintilimab treatment, characterized by systemic immune hyperactivation and likely pre-existing skin barrier impairment through mechanisms noted earlier, externally derived limonene carboxylic acid—potentially from dietary citrus, personal care products, or fragrances—may permeate the skin and function as a hapten. By forming neoantigens via covalent binding to cutaneous proteins, it can be internalized by Langerhans cells and presented to PD-1-inhibitor-primed T cells, thereby provoking a strong allergic contact dermatitis-like reaction (Nishijima et al., 1997). Conversely, endogenously produced limonene carboxylic acid may be upregulated as a compensatory anti-inflammatory mediator in an attempt to counterregulate localized inflammation; however, its efficacy is likely overwhelmed in the context of robust T cell-mediated skin injury. Collectively, the emergence of limonene carboxylic acid underscores a critical interaction between exogenous environmental exposure and endogenous immune sensitization, supporting its potential involvement in the pathogenesis and exacerbation of sintilimab-induced skin rash.

N-lauroylsarcosine, identified as another differential metabolite in this study, is an anionic surfactant formed through the condensation of lauric acid and sarcosine (N-methylglycine). Its aberrant accumulation likely stems from sintilimab-induced systemic inflammation, wherein pro-inflammatory cytokines suppress key epidermal lipid metabolic enzymes, disrupt fatty acid homeostasis—including lauric acid metabolism—and perturb one-carbon and methylation pathways (Morellato et al., 2021; Yan et al., 2022). Possessing strong lipid- and protein-dissolving properties, N-lauroylsarcosine destabilizes cell membranes and impairs stratum corneum bilayer integrity. Even at endogenously low concentrations, it acts similarly to exogenous surfactants by compromising skin barrier function through physicochemical mechanisms, resulting in excessive lipid removal, elevated transepidermal water loss, and enhanced penetration of external irritants (Kim et al., 2008). This sustained barrier dysfunction perpetuates “danger signals” that activate innate immune pathways—such as Toll-like receptor cascades—promote inflammatory cytokine secretion, and amplify T-cell-driven immune responses, thereby establishing a self-renewing cycle of inflammation and barrier failure (Wong et al., 2009). Additionally, its inherent antimicrobial activity may disrupt the cutaneous microbiota and alter the local immune microenvironment (Zhao et al., 2024). In contrast to alkaline surfactants like sodium laurate, N-lauroylsarcosine is mildly acidic and helps maintain native skin surface pH, thereby avoiding delayed pruritus, though it can still provoke acute mechanical irritation (Inami et al., 2013). Importantly, this metabolite may also arise through microbial metabolism of drug excipients: microbial esterases could hydrolyze polysorbate 80 in the sintilimab formulation to release lauric acid, which may then conjugate with host-derived sarcosine, highlighting a potential drug–microbiome–host interplay in immune-related dermal toxicity. Thus, N-lauroylsarcosine not only serves as a biomarker of disturbed epidermal lipid metabolism but may also play an active role in the initiation and persistence of sintilimab-associated rash via direct barrier disruption and potentiation of innate immunity.

It should be noted that the endogenous or dietary/environmental origin of these metabolites was inferred based on database annotations and literature rather than confirmed by experiments; future studies with stable isotope tracing or controlled dietary interventions would help verify their precise origins. Additionally, all of these top metabolites were among the differential metabolites used for KEGG enrichment, and each is directly or indirectly involved in the top pathways discussed above (e.g., ergothioneine in FcεRI signaling, laurolactam in CoA biosynthesis). Collectively, the above mechanistic interpretations of the five key metabolites are hypothetical and exploratory, and further *in vitro*, *in vivo*, or clinical experiments are warranted to verify their exact roles in sintilimab-induced rash.

While the metabolomic profiles generated in this study offer valuable mechanistic insights into the pathogenesis of sintilimab-associated cutaneous toxicity, several critical limitations need to be acknowledged. Primarily, our findings are preliminary due to the small sample size and absence of an independent external validation cohort in this single-center study, which markedly limits the generalizability of the identified biomarker panel. As such, these results are hypothesis-generating in nature and mandate further verification in independent patient populations. The top differentially expressed metabolites screened via univariate analysis and machine learning algorithms should be prioritized for targeted quantitative validation using orthogonal methodologies (e.g., MRM LC-MS/MS) in large-scale, multicenter cohorts. In addition, although our pathway enrichment analysis was based on dysregulated metabolites, direct statistical correlations between individual key metabolites and the activity scores of top enriched pathways were not assessed in the current study; this correlation analysis is a crucial priority for subsequent large-cohort investigations to clarify the mechanistic links between metabolic dysregulation and cutaneous toxicity. Finally, exclusive reliance on plasma biospecimens narrows the metabolic scope of this study; future research should prioritize multi-compartment metabolic profiling (e.g., skin interstitial fluid, skin tissue biopsies) to establish a comprehensive multi-omics atlas of immune checkpoint inhibitor (ICI)-induced adverse events. Looking forward, the candidate metabolite panel identified herein holds clinical translational potential for two core applications: (i) pre-treatment risk stratification, whereby baseline metabolite levels could be measured to identify high-risk patients requiring enhanced dermatological surveillance; and (ii) early pharmacodynamic monitoring, via serial metabolite testing during the first 1–2 treatment cycles to detect metabolic perturbations prior to the clinical onset of rash. Prospective multicenter validation in independent cohorts is indispensable prior to clinical implementation of this biomarker panel. Furthermore, future integration of pharmacometabolomics with genome-scale functional module analysis will refine the accuracy and translational value of predictive models for sintilimab-induced rash (Tian et al., 2025b).

## 5 Conclusion

This study utilized untargeted metabolomics to characterize metabolic perturbations associated with sintilimab-induced cutaneous toxicity in lung cancer patients. Our findings implicate dysregulated immune and inflammatory responses in the pathogenesis of immune-mediated (IM) skin rashes. We identified several key metabolites with strong potential as predictive biomarkers for rash development. These included N,N,N-trimethyl-L-histidine, laurolactam, 2-naphthalenesulfonic acid, limonenecarboxylic acid, and N-lauroylsarcosine. Collectively, these results provide novel insights into the pathological basis of sintilimab-associated dermatological adverse events. The identified metabolite signature may inform future therapeutic strategies for managing this toxicity. Further validation through larger, multi-center cohorts and mechanistic studies is required to confirm these metabolic pathway aberrations and establish clinical utility. If confirmed, the five overlapping metabolites could support pre-treatment risk stratification and early pharmacodynamic monitoring to minimize treatment disruptions.

## Data Availability

The original contributions presented in the study are publicly available. his data can be found in the Figshare repository (accession number: 10.6084/m9.figshare.32398785; available at: https://figshare.com/s/92f18bdfa6f6c0eddcb2).

## Supplementary material

SUPPLEMENTARY FIGURE S1 PCA score plot of the overall sample in positive mode detection **(A)** and negative mode detection **(B)**. The red dots represent QC samples, while the colored dots indicate the actual samples.

SUPPLEMENTARY FIGURE S1 Correlation heatmap of significantly altered metabolites. The heatmap depicts pairwise correlation patterns among differentially abundant metabolites. Both the vertical axis and horizontal axis represent the names of differential metabolites. Color gradient indicates Pearson correlation coefficients, with deep red signifying strong positive correlations (r → +1) and deep blue denoting strong negative correlations (r → −1). Color intensity scales proportionally with the absolute correlation strength.

SUPPLEMENTARY TABLE S1 Differential metabolites identified in positive mode and negative mode.

SUPPLEMENTARY TABLE S2 ROC analysis of serum differential metabolites between control group and rash group.
