# Supplementary material for: Integrated metabolomics and machine learning identify predictive biomarkers via SHAP analysis for sintilimab-induced rash in lung cancer patients
Source: Front Pharmacol. 2026 Jun 3;17:1846667. doi: 10.3389/fphar.2026.1846667 (PMC13272154; doi:10.3389/fphar.2026.1846667)
Supplement: Supplementary file 1 [file Table1.docx]

**Table S1 Differential metabolites identified in positive mode and negative mode.**

| **precursor type** | **name** | **VIP** | **m/z** | **rt** | **FC** | **log2FC** | **P.value** | **Pos/neg** |
| --- | --- | --- | --- | --- | --- | --- | --- | --- |
| [M-H]- | (+)-Diacetyl-L-tartaric anhydride | 2.75317 | 215.0227 | 244 | 0.54 | -0.89 | 3E-07 | neg |
| [M+Na]+ | (1S,4S,5R,6S,8R,16S,19S,21S)-14-ethyl-4,6,19-trimethoxy-16-(methoxymethyl)-9,11-dioxa-14-azaheptacyclo[10.7.2.12,5.01,13.03,8.08,12.016,20]docosan-21-ol | 2.307476 | 502.2766 | 243.989 | 0.59 | -0.77 | 0.000159 | pos |
| [M+H]+ | (4S)-7-Hydroxy-4-isopropenyl-7-methyloxepan-2-one | 2.527314 | 185.115 | 42.8636 | 1.13 | 0.17 | 8.09E-05 | pos |
| [M+H]+ | [4,4,6a,6b,8a,11,14b-heptamethyl-14-oxo-11-(pyridin-2-ylcarbamoyl)-2,3,4a,5,6,7,8,9,10,12,12a,14a-dodecahydro-1H-picen-3-yl] acetate | 2.227906 | 589.4078 | 408.064 | 0.44 | -1.19 | 0.000292 | pos |
| [M+NH4]+ | 10-Apo-beta-carotenal | 2.072195 | 394.31a58 | 432.277 | 1.29 | 0.37 | 0.006898 | pos |
| [M+H]+ | 1-Hydroxy-2,5-dioxopyrrolidine-3-sulfonic acid | 3.179234 | 195.9931 | 33.5236 | 0.88 | -0.19 | 1.05E-08 | pos |
| [M-H]- | 2,3,4,6-Tetrahydroxybenzophenone | 2.382279 | 245.044 | 294.269 | 0.63 | -0.67 | 0.000102 | neg |
| [M+H]+ | 2,4-Dibromo-1-(4-bromophenoxy)benzene | 2.229187 | 404.816 | 33.62355 | 0.71 | -0.49 | 3.59E-05 | pos |
| [M+H]+ | 2,4-Dichlorophenol | 2.283158 | 163.9396 | 34.7885 | 0.87 | -0.21 | 7.3E-05 | pos |
| [M-H]- | 2,4-Dinitrophenol | 2.203651 | 183.0034 | 109.643 | 1.35 | 0.44 | 0.000223 | neg |
| [M+NH4]+ | 2,5-dichloromuconic acid | 2.482646 | 227.9854 | 33.31775 | 0.86 | -0.22 | 0.000122 | pos |
| [M+Na]+ | 26-hydroxybrassinolide | 3.07495 | 519.3268 | 350.8125 | 4.49 | 2.17 | 3.29E-07 | pos |
| [M+NH4]+ | 2-Hexen-1-ol | 2.311397 | 118.1229 | 55.6769 | 1.52 | 0.61 | 0.000127 | pos |
| [M-H]- | 2-Isopropyl-3,5-dimethoxy-6-methylpyrazine | 2.527509 | 195.1142 | 132.193 | 1.79 | 0.84 | 7.88E-06 | neg |
| [M-H]- | 2-Naphthalenesulfonic acid | 2.240987 | 207.0126 | 230.003 | 1.95 | 0.96 | 0.000114 | neg |
| [M+Na]+ | 2-Stearoyl-sn-glycero-3-phosphocholine | 2.233765 | 546.3472 | 337.9845 | 2.73 | 1.45 | 0.000243 | pos |
| [M-CO2+H]+ | 2-Thio-PAF | 2.415906 | 496.361 | 350.967 | 3.4 | 1.76 | 0.000139 | pos |
| [M+NH4]+ | 3-(4-Hydroxyphenyl)-1-propanol | 2.611592 | 170.1179 | 422.99 | 1.55 | 0.63 | 3.4E-05 | pos |
| [M+H]+ | 3-(Diethylamino)-1-(3,4-dimethoxyphenyl)propan-1-one | 2.145808 | 266.1726 | 303.643 | 2.23 | 1.15 | 6.67E-05 | pos |
| [M+CH3CO2]- | 3,6-Dichloropyridine-2,5-dione | 2.022066 | 235.9555 | 34.0445 | 0.53 | -0.92 | 0.000448 | neg |
| [M-NH3+H]+ | 3-Chlorophenol | 2.726449 | 111.9845 | 33.52925 | 0.82 | -0.28 | 9.73E-07 | pos |
| [M+HCOO]- | 3-deoxy-D-arabino-heptulosonate-7-phosphate | 2.366979 | 333.0226 | 298.37 | 0.73 | -0.46 | 8.54E-06 | neg |
| [M-H]- | 3-Hydroxy-L-tyrosyl-AMP | 2.238876 | 525.1198 | 357.976 | 0.66 | -0.59 | 0.00032 | neg |
| [M-NH3+H]+ | 4,4-Diaponeurosporen-4-al | 2.064739 | 400.293 | 406.756 | 0.65 | -0.61 | 0.00194 | pos |
| [M-H2O-H]- | 4,6-Dichloro-3-methylcatechol | 2.091054 | 172.9571 | 74.89985 | 0.72 | -0.47 | 4.28E-05 | neg |
| [M+H]+ | 4alpha-hydroxymethyl-ergosta-7,24(241)-dien-3beta-ol | 2.356991 | 429.3677 | 403.358 | 2.91 | 1.54 | 0.000336 | pos |
| [M+Na]+ | 4-Hydroxy-6-methyl-2-pyrone | 3.234499 | 149.0229 | 35.03265 | 0.9 | -0.14 | 7.97E-09 | pos |
| [M-NH3-H]- | 4-Hydroxyestradiol | 2.748523 | 270.1353 | 283.245 | 1.58 | 0.66 | 5.31E-08 | neg |
| [M-H]- | 5-dimethylamiloride | 2.759095 | 256.0731 | 292.745 | 0.5 | -0.99 | 6.34E-07 | neg |
| [M+NH4]+ | 7-hydroxy-1-({3-o-[(2e)-8-hydroxy-2,6-dimethyl-2-octenoyl]-beta-d-glucopyranosyl}oxy)-7-methyl-1,4a,5,6,7,7a-hexahydrocyclopenta[c]pyran-4-carboxylic acid | 2.443874 | 562.2863 | 367.929 | 0.7 | -0.5 | 2.12E-05 | pos |
| [M+HCOO]- | 7-Methylguanosine 5'-phosphate | 2.321831 | 423.0749 | 408.45 | 0.68 | -0.55 | 1.43E-05 | neg |
| [M-H]- | 7-Methylxanthine | 2.379417 | 165.0411 | 407.74 | 0.85 | -0.23 | 1.72E-05 | neg |
| [M+H]+ | Adenosine 5'-tetraphosphate(5-) | 2.150467 | 582.9253 | 49.6742 | 1.38 | 0.46 | 0.000563 | pos |
| [M-H]- | Anthraquinone | 2.326967 | 207.0473 | 259.909 | 0.34 | -1.54 | 6.86E-05 | neg |
| [M+H]+ | Asn-Phe | 2.678975 | 280.1326 | 238.362 | 0.47 | -1.1 | 8.26E-06 | pos |
| [M-H2O+H]+ | Auranofin | 2.097245 | 661.1266 | 392.2735 | 0.55 | -0.86 | 0.000687 | pos |
| [M+Na]+ | Austrobuxusin I | 2.198991 | 613.3327 | 245.088 | 0.69 | -0.53 | 0.000385 | pos |
| [M+Na]+ | CID 24121299 | 2.823231 | 683.3761 | 449.0585 | 2.85 | 1.51 | 5.38E-06 | pos |
| [M+K]+ | Coproporphyrinogen III | 2.077057 | 699.4082 | 241.543 | 0.56 | -0.84 | 0.00049 | pos |
| [M+H]+ | Cryptotanshinone | 2.12941 | 297.1468 | 348.978 | 2.23 | 1.16 | 0.000962 | pos |
| [M+H]+ | Cyclomytiloxanthin | 2.184695 | 617.4286 | 437.074 | 0.57 | -0.8 | 0.000539 | pos |
| [M-H]- | DG(14_1(9Z)_18_4(6Z,9Z,12Z,15Z)_0_0) | 2.037491 | 557.4166 | 415.9235 | 1.74 | 0.8 | 0.000228 | neg |
| [M-H]- | Di-Ac- Psammaplysin A | 2.032331 | 811.8458 | 58.5715 | 1.9 | 0.92 | 4.23E-05 | neg |
| [M+H]+ | Dihydrocaffeic acid 3-sulfate | 2.746783 | 263.0247 | 72.5746 | 0.62 | -0.68 | 1.2E-05 | pos |
| [M-H2O+H]+ | Dihydroxyacetone phosphate | 2.695289 | 152.9951 | 33.6313 | 0.81 | -0.31 | 3.73E-06 | pos |
| [M+H]+ | Drofenine | 2.02028 | 318.2407 | 394.019 | 0.61 | -0.71 | 0.001652 | pos |
| [M+HCOO]- | dUDP | 3.094665 | 433.0102 | 226.194 | 0.37 | -1.44 | 2.29E-09 | neg |
| [M+NH4]+ | Ergosterol | 2.110587 | 414.376 | 425.833 | 2.09 | 1.06 | 0.000555 | pos |
| [M-NH3+H]+ | Erythrulose 1-phosphate | 3.061967 | 183.9897 | 34.1565 | 0.78 | -0.36 | 1.75E-07 | pos |
| [M+Na]+ | Ethylene diamine tetra (methylene phosphonic acid) | 2.044235 | 778.8488 | 46.9621 | 0.8 | -0.32 | 0.000904 | pos |
| [M+H]+ | FA 14_1 | 2.242112 | 227.201 | 371.3315 | 1.37 | 0.46 | 0.000198 | pos |
| [M+H]+ | gamma-Sitosterol | 2.505131 | 415.3883 | 405.255 | 1.74 | 0.8 | 4.17E-05 | pos |
| [M+H]+ | Indoleacetic acid | 2.026317 | 176.0708 | 279.046 | 0.7 | -0.5 | 0.001257 | pos |
| [M+H]+ | Isokobusone | 2.328167 | 223.1697 | 394.6085 | 1.26 | 0.34 | 3.37E-05 | pos |
| [M+Na]+ | Janthitrem C | 2.477312 | 592.3343 | 424.3375 | 1.69 | 0.76 | 0.000115 | pos |
| [M+HCOO]- | Kadsulignan N | 2.409713 | 475.1951 | 352.8605 | 1.4 | 0.48 | 2.61E-05 | neg |
| [M+Na]+ | Karaviloside V | 2.476012 | 835.4848 | 247.393 | 0.6 | -0.75 | 1.35E-05 | pos |
| [M+NH4]+ | L-765,314 hydrate | 3.174151 | 558.3018 | 244.513 | 0.52 | -0.93 | 4.38E-09 | pos |
| [M-CO2+H]+ | Lacinilene C | 2.432986 | 203.1432 | 406.42 | 1.18 | 0.24 | 0.000237 | pos |
| [M+H]+ | Laurolactam | 2.681298 | 198.1855 | 303.357 | 1.59 | 0.67 | 1.12E-06 | pos |
| [M+NH4]+ | Limonenecarboxylic acid | 2.688972 | 198.1494 | 274.273 | 1.5 | 0.58 | 1.84E-06 | pos |
| [M-H]- | L-Tartaric acid | 2.062247 | 149.0099 | 407.716 | 0.87 | -0.21 | 0.000204 | neg |
| [M-H2O+H]+ | L-threo-sphinganine | 2.078322 | 285.2985 | 411.333 | 0.53 | -0.9 | 0.000368 | pos |
| [M+NH4]+ | LysoPC(15_0_0_0) | 2.42091 | 499.3469 | 350.665 | 3.29 | 1.72 | 0.000248 | pos |
| [M+HCOO]- | Mesylate | 2.071011 | 140.9879 | 37.9519 | 0.67 | -0.57 | 0.00023 | neg |
| [M+CH3CO2]- | Methyl 1-(1-propenylsulfinyl)propyl disulfide | 2.123483 | 269.0342 | 280.232 | 1.46 | 0.55 | 0.000197 | neg |
| [M+NH4]+ | Metoclopramide | 2.538641 | 317.6594 | 349.3125 | 2.61 | 1.38 | 7.98E-05 | pos |
| [M+H]+ | Myrtucommulone A | 2.406679 | 669.3662 | 245.65 | 0.62 | -0.69 | 1.16E-05 | pos |
| [M+HCOO]- | N,N,N-Trimethyl-Histidine | 2.407783 | 243.1239 | 241.982 | 1.77 | 0.82 | 1.15E-05 | neg |
| [M-H]- | N-Acetylneuraminic acid | 2.076285 | 308.0987 | 46.5177 | 0.56 | -0.84 | 0.000378 | neg |
| [M+NH4]+ | NCGC00380376-01_C22H40O3_1-Naphthalenepentanol, decahydro-2-hydroxy-gamma,2,5,5,8a-pentamethyl-, alpha-acetate | 2.477438 | 370.3312 | 406.421 | 1.75 | 0.8 | 3.72E-05 | pos |
| [M+NH4]+ | N-Hydroxy-3,4,5-trimethoxybenzamide | 2.490441 | 245.1116 | 236.2305 | 0.5 | -1 | 4.31E-05 | pos |
| [M-H]- | N-Lauroylsarcosine | 2.177558 | 270.2075 | 280.869 | 1.68 | 0.75 | 0.000141 | neg |
| [M+Na]+ | N-Methyl-4-dimethylallyltryptophan | 2.911797 | 309.1529 | 235.09 | 0.48 | -1.05 | 3.26E-07 | pos |
| [M+H]+ | Pantetheine | 2.993396 | 279.135 | 238.362 | 0.41 | -1.28 | 2.36E-07 | pos |
| [M+H]+ | Parecoxib | 2.331692 | 371.102 | 440.156 | 1.42 | 0.51 | 0.000513 | pos |
| [M-H2O-H]- | PC(18_1_8,9-EpETE) | 2.108578 | 802.5487 | 318.142 | 1.79 | 0.84 | 0.000146 | neg |
| [M-H2O-H]- | PC(18_1_8-Hepe) | 2.041592 | 802.5504 | 335.344 | 2.02 | 1.01 | 0.000269 | neg |
| [M-H2O+H]+ | Peridinin | 2.950268 | 613.3466 | 245.372 | 0.48 | -1.06 | 3.02E-07 | pos |
| [M-H2O+H]+ | Phosphoenol-4-deoxy-3-tetrulosonate | 2.720639 | 180.9892 | 33.8792 | 0.83 | -0.27 | 8.13E-07 | pos |
| [M-H]- | Plumbagin | 2.265077 | 187.0423 | 49.9975 | 0.53 | -0.92 | 0.000131 | neg |
| [M+H]+ | Pravastatin Sodium | 3.14918 | 447.2357 | 243.35 | 0.51 | -0.96 | 4.51E-09 | pos |
| [M-NH3-H]- | Pretetramid | 2.022341 | 333.0407 | 102.181 | 1.85 | 0.89 | 0.001401 | neg |
| [2M-H]- | Pyridoxine 5'-phosphate | 2.234338 | 497.0782 | 384.8355 | 0.48 | -1.06 | 0.000237 | neg |
| [M+H]+ | ST 29_2;O | 2.112661 | 413.3727 | 425.726 | 1.68 | 0.75 | 0.000493 | pos |
| [M+H]+ | Stercobilin | 2.223212 | 595.3532 | 248.971 | 0.68 | -0.56 | 0.000588 | pos |
| [M+H]+ | Suberate | 2.894068 | 173.0787 | 59.141 | 0.66 | -0.6 | 4.12E-07 | pos |
| [M+NH4]+ | Theionbrunonine A | 2.083751 | 724.3977 | 246.238 | 0.67 | -0.58 | 0.000576 | pos |
| [M-H2O+H]+ | trans-2-Chlorodienelactone | 2.31095 | 156.9676 | 33.4886 | 0.82 | -0.28 | 2.85E-05 | pos |
| [M-CO2+H]+ | Tricrocin | 2.237197 | 771.3425 | 46.7388 | 1.4 | 0.49 | 0.000503 | pos |
| [M+NH4]+ | Vitamin K | 2.613144 | 468.3864 | 441.085 | 0.52 | -0.95 | 3.91E-05 | pos |
| [M+H]+ | Zenkerine | 2.137754 | 298.144 | 349.0165 | 2.55 | 1.35 | 0.000383 | pos |
