# Supplementary figures and images for: Integrated metabolomics and machine learning identify predictive biomarkers via SHAP analysis for sintilimab-induced rash in lung cancer patients
Source: Front Pharmacol. 2026 Jun 3;17:1846667. doi: 10.3389/fphar.2026.1846667 (PMC13272154; doi:10.3389/fphar.2026.1846667)

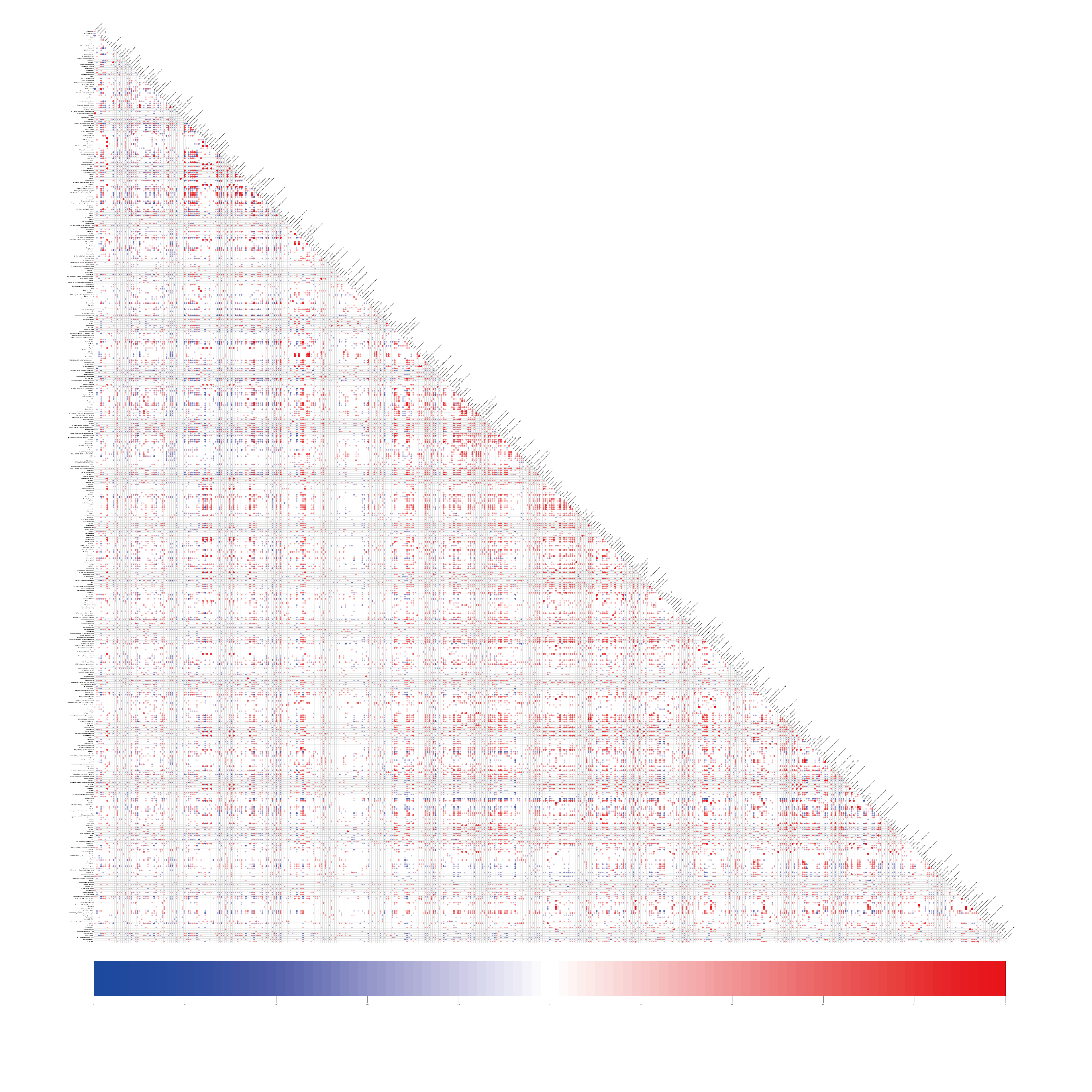

Supplement: Supplementary file 2 [file Image2.tif]

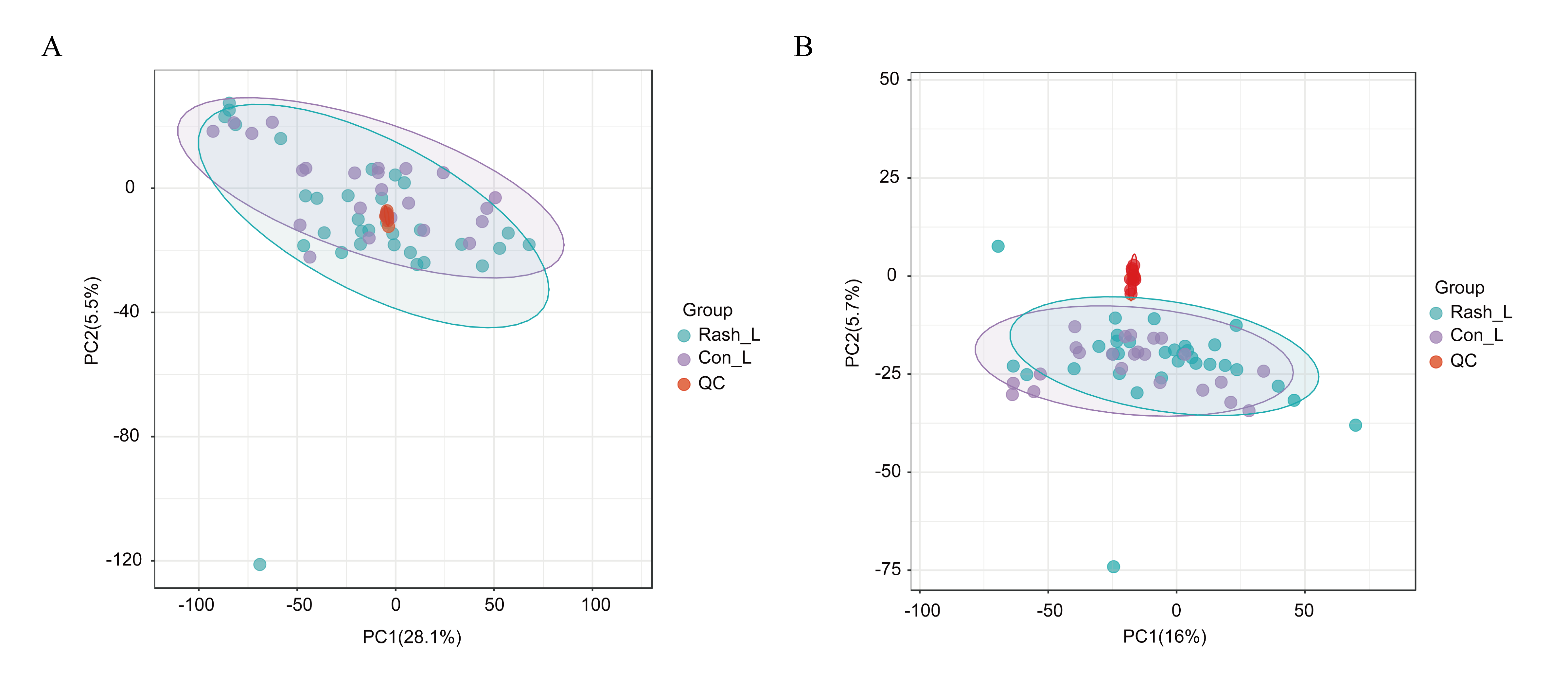

Supplement: Supplementary file 3 [file Image1.tif]
