# Supplementary material for: Integrated metabolomics and machine learning identify predictive biomarkers via SHAP analysis for sintilimab-induced rash in lung cancer patients
Source: Front Pharmacol. 2026 Jun 3;17:1846667. doi: 10.3389/fphar.2026.1846667 (PMC13272154; doi:10.3389/fphar.2026.1846667)
Supplement: Supplementary file 4 [file Table2.docx]

**Table S2** ROC analysis of serum differential metabolites between control group and rash group.

| name | AUC_value | 95% CI |
| --- | --- | --- |
| 26-hydroxybrassinolide | 0.879 | [0.773, 0.959] |
| CID 24121299 | 0.878 | [0.768, 0.965] |
| Laurolactam | 0.875 | [0.769, 0.956] |
| 4-Hydroxyestradiol | 0.871 | [0.764, 0.952] |
| 3-(4-Hydroxyphenyl)-1-propanol | 0.859 | [0.758, 0.948] |
| 2-Naphthalenesulfonic acid | 0.849 | [0.735, 0.949] |
| 4-Chloro-5-sulfamoylanthranilic acid | 0.846 | [0.731, 0.943] |
| Sudan_IV | 0.842 | [0.715, 0.949] |
| Limonenecarboxylic acid | 0.841 | [0.724, 0.943] |
| 4-Aminocatechol | 0.840 | [0.718, 0.951] |
| 2-Isopropyl-3,5-dimethoxy-6-methylpyrazine | 0.838 | [0.734, 0.934] |
| N,N,N-Trimethyl-Histidine | 0.837 | [0.720, 0.946] |
| Pyrrhoxanthinol | 0.832 | [0.721, 0.923] |
| 2-Stearoyl-sn-glycero-3-phosphocholine | 0.829 | [0.693, 0.930] |
| Janthitrem C | 0.827 | [0.720, 0.923] |
| Isokobusone | 0.823 | [0.704, 0.921] |
| PC(18_1_8,9-EpETE) | 0.821 | [0.682, 0.931] |
| (4S)-7-Hydroxy-4-isopropenyl-7-methyloxepan-2-one | 0.819 | [0.692, 0.935] |
| NCGC00380376-……-pentamethyl-,alpha-acetate | 0.813 | [0.691, 0.917] |
| Metoclopramide | 0.807 | [0.670, 0.919] |
| gamma-Sitosterol | 0.804 | [0.676, 0.909] |
| pyrethrin I | 0.800 | [0.673, 0.909] |
| Kadsulignan N | 0.800 | [0.685, 0.904] |
| FA 14_1 | 0.800 | [0.669, 0.905] |
| N-Lauroylsarcosine | 0.800 | [0.669, 0.904] |

**Abbreviations:** AUC: Area under the curve.
